# Peripandemic psychiatric emergencies: impact of the COVID-19 pandemic on patients according to diagnostic subgroup

**DOI:** 10.1007/s00406-020-01228-6

**Published:** 2021-02-05

**Authors:** Johanna Seifert, Catharina Meissner, Anna Birkenstock, Stefan Bleich, Sermin Toto, Christian Ihlefeld, Tristan Zindler

**Affiliations:** Department of Psychiatry, Social Psychiatry, and Psychotherapy, Hanover Medical School, Hanover, Germany

**Keywords:** Psychiatric emergency department, COVID-19, Mental health, Pandemic, Psychological burden

## Abstract

On March 11th, 2020, the outbreak of coronavirus disease 2019 (COVID-19) was declared a pandemic. Governments took drastic measures in an effort to reduce transmission rates and virus-associated morbidity. This study aims to present the immediate effects of the pandemic on patients presenting in the psychiatric emergency department (PED) of Hannover Medical School. Patients presenting during the same timeframe in 2019 served as a control group. A decrease in PED visits was observed during the COVID-19 pandemic with an increase in repeat visits within 1 month (30.2 vs. 20.4%, *p*_BA_ = 0.001). Fewer patients with affective disorders utilized the PED (15.2 vs. 22.2%, *p*_BA_ = 0.010). Suicidal ideation was stated more frequently among patients suffering from substance use disorders (47.4 vs. 26.8%, *p*_BA_ = 0.004), while patients with schizophrenia more commonly had persecutory delusions (68.7 vs. 43.5%, *p*_BA_ = 0.023) and visual hallucinations (18.6 vs. 3.3%, *p*_BA_ = 0.011). Presentation rate of patients with neurotic, stress-related, and somatoform disorders increased. These patients were more likely to be male (48.6 vs. 28.9%, *p*_BA_ = 0.060) and without previous psychiatric treatment (55.7 vs. 36.8%, *p*_BA_ = 0.089). Patients with personality/behavioral disorders were more often inhabitants of psychiatric residencies (43.5 vs. 10.8%, *p*_BA_ = 0.008). 20.1% of patients stated an association between psychological well-being and COVID-19. Most often patients suffered from the consequences pertaining to social measures or changes within the medical care system. By understanding how patients react to such a crisis situation, we can consider how to improve care for patients in the future and which measures need to be taken to protect these particularly vulnerable patients.

**Supplementary Information:**

The online version contains supplementary material available at 10.1007/s00406-020-01228-6.

## Introduction

December 2019 marked the beginning of the coronavirus disease 2019 (COVID-19), which, by March 11th, 2020, had officially been declared a pandemic according to the World Health Organization [1]. In hopes of stalling the spread of the virus, governments began taking drastic measures: schools, office buildings, restaurants, stores selling “non-essential” items (e.g. clothing, jewelry, books), along with international borders, and entire economies were shut down. “Social distancing” measures were implemented instructing citizens to leave their homes only when truly necessary, while others were quarantined due to a suspected case of COVID-19 or after having been in contact with a confirmed case of COVID-19. With overwhelming attention paid to the adverse health outcomes directly resulting from this disease, it is important not to lose sight of other potential negative effects on public health. This concern especially applies to mental health [2], and in particular to those who already suffer from mental illness [2–4]. An association between viral epidemics and a decrease in mental health was first documented over 100 years ago when American psychiatrist Karl Augustus Menninger described a link between the Spanish flu pandemic in 1918 with psychiatric morbidity [5]. While emergency containment measures may help slow the spread of the virus, they have a major impact on daily life, potentially resulting in an increased psychological burden [6]. The aim of this study was to evaluate in which aspects (e.g. psychological aspects, diagnosis, gender, age, time, and means of presentation, etc.) patients from different diagnostic subgroups seeking emergency psychiatric care during the COVID-19 pandemic differed from patients who presented in the psychiatric emergency department (PED) in the previous year during the same timeframe.

## Methods

### Data collection

Hannover Medical School (German: Medizinische Hochschule Hannover, MHH) is an academic teaching hospital and one of four psychiatric hospitals in Hannover and its municipals servicing a catchment area of 138,471 residents. MHH is the only department of psychiatry within Hannover’s city limits and the only psychiatric department with other medical disciplines (e.g. internal medicine, neurology) on site. The PED is visited by over 2500 patients annually. Electronic documentation of all patients seeking emergency psychiatric care at in the PED of MHH from March 16th to May 24th, 2020 was collected by three psychiatric residents. All patients aged 18 years and older were included in data collection. Patients leaving the PED prior to contact with the psychiatric resident on call were excluded from further analysis. Apart from the physicians taking part in data collection, all other psychiatrists on call were unaware of this study and had not been previously instructed to ask patients how the current situation was affecting their state of mental health.

Electronic patient documentation was used to extract relevant information including basic sociodemographic and clinical characteristics as well as last treatment within the department of psychiatry of MHH (including inpatient treatment and emergency consultation). Primary psychiatric diagnosis was documented according to the International Classification of Disease in its 10th Version (ICD-10) [7] and then grouped according to major diagnosis subgroup (F1–F4 and F6). Diagnoses not falling within these subgroups (i.e. F0, F5, and F7–9) were classified as “others”. All data was de-identified by pseudonymization.

Further, 65 individual aspects of the psychopathological assessment (PPA) according to the “Arbeitsgemeinschaft für Methodik und Dokumentation in der Psychiatrie” (AMDP)-System [8], routinely documented for each patient, were assessed. The AMDP-System is a manual for standardized documentation of PPA commonly used in German-speaking countries. It consists of a glossary of psychopathological symptom descriptions pertaining to different aspects of PPA. In this study, aspects of PPA considered highly relevant for emergency psychiatric care (e.g. orientation, formal and content thought disorders, affective disturbances, suicidality) [8] that were therefore expected to be reliably documented were extracted by looking for key words as predetermined by the AMDP-Manual.

### Determination of the time period for data collection

**Fig. 1:**
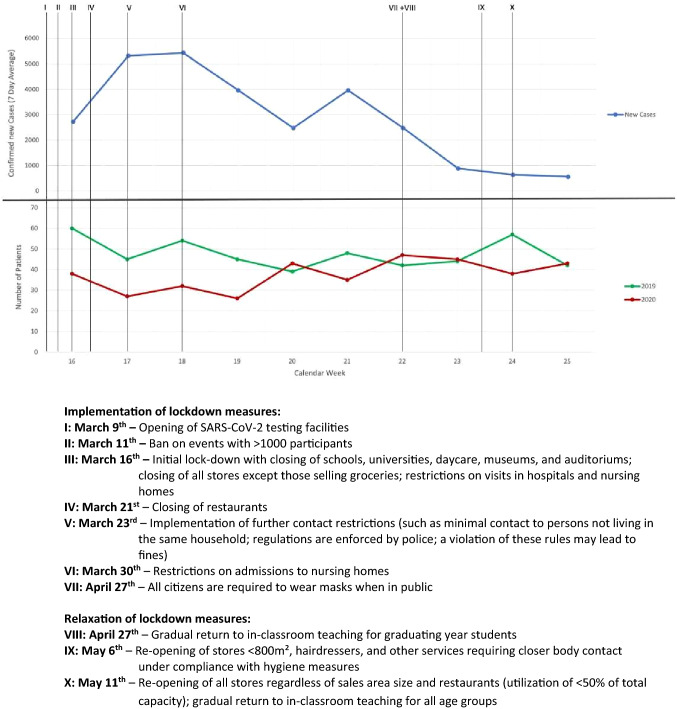
Number of newly confirmed SARS-CoV-2 cases, number of patients presenting in the PED (2020 vs. 2019), and implementation/relaxation of lockdown measures. *SARS-CoV-2* severe acute respiratory syndrome coronavirus 2, *PED* psychiatric emergency department

March 16th, 2020 was selected as starting point as this was the day that marked the closing of all schools, stores selling non-essential goods, and recreational facilities in the state of Lower Saxony, Germany. Data collection ended on May 24th, 2020 at which time restaurants, selected recreational locations, and schools for older students had re-opened but other “social distancing” measures were still in place. Figure 1 gives a further overview of measures taken in Lower Saxony to stall the spread of COVID-19 as well as the first steps in easing lockdown measures. Patients presenting for emergency psychiatric consultation during the same time period (March 16th–May 24th) in 2019 served as a control group.

### Association with COVID-19

An association between the current circumstances surrounding the COVID-19 pandemic and its influence on a patient’s mental health was noted only if it had been explicitly documented by the psychiatrist on call. The impact of COVID-19 on mental health was categorized as follows:

- psychological consequences of social measures such as “social distancing”, quarantine, restrictions on leaving the house and visiting friends/family (e.g. isolation, suffering due to these restrictions, conflicts with family members resulting from spending more time confined within the same space)
- changes in the structures of medical care (e.g. unavailability of sufficient outpatient treatment, re-presentation after premature discharge due to “emergency only” inpatient treatment)
- delusional content and/or hallucinations pertaining to the coronavirus, pandemic and/or social measures (e.g. persecutory delusions, delusion of guilt, delusion of reference)
- anxiety, fear, and/or compulsive behavior (e.g. fear of infection, fear of transmission, compulsive disinfection and/or handwashing)
- loss of job in context with the pandemic and its psychological consequences
- “others” (e.g. shortage of drugs, increased work-load associated with the pandemic, acute intoxication with alcohol for its disinfecting properties).

### Statistical methods

Besides the presented descriptive data, inferential statistical group differences were calculated with a series of Chi-square tests. Because of the high number of comparisons, corrections were made for multiple testing. The Bonferroni adjusted level of significance was set to *p*_BA_ < 0.05 for each of the subsections. Accordingly, the Bonferroni adjustments were made for each of the subsections as shown in the supplementary material Tables 1–5. To improve readability of the manuscript, Table 4 only shows significant findings made within the diagnostic subgroups.

### Ethical approval

Ethical approval for this study was obtained from the Clinical Ethics Committee of Hannover Medical School (No. 9058_BO_K_2020). The investigation was carried out in accordance with the latest version of the Declaration of Helsinki.

## Results

Between March 16th and May 24th 2020, a total of 392 patients registered for emergency psychiatric consultation of which 18 (4.6%) left prior to seeing a physician. During the same time period in 2019, 488 patients came to the PED, 12 (2.5%) leaving before consultation. The study sample referred to in the following does not include the patients leaving prior to treatment.

### Study sample characteristics

Overall, fewer patients utilized emergency psychiatric care during the COVID-19 pandemic than in the previous year (*n*_2020_ = 374 vs. *n*_2019_ = 476 patients; relative decrease of 21.4%). This was largely due to a decrease between March 16th and April 12th (Fig. 1). Both in 2020 and in 2019, more than half of patients presenting in the PED were male (60.7 vs. 52.1%, *X*^2^ (1, *N* = 850) = 6.275, *p* = 0.012, *p*_BA_ > 0.135). By trend, during the COVID-19 pandemic, patients were more likely to come to the PED during “off-hours” (i.e. between 20:00 and 07:59) than in the year prior (35.3 vs. 26.7%, *X*^2^ (1, N = 850) = 7.334, *p* = 0.007, *p*_BA_ = 0.074) and also less likely to be admitted to inpatient care (52.1 vs. 61.6%, *X*^2^ (1, *N* = 850) = 7.594, *p* = 0.006, *p*_BA_ = 0.064). A significantly higher number of repeat visits to the PED within 1 month was observed in 2020 (30.2 vs. 20.4%, *X*^2^ (1, *N* = 850) = 10.892, *p* = 0.001, *p*_BA_ = 0.011). The variables “age”, “legal basis of psychiatric treatment”, “means of presentation”, “attempted suicide prior to presentation”, and “previous psychiatric treatment” remained unchanged (Table 1).

**Table 1:** General characteristics of the study population in 2020 vs. 2019

	2020	2019	chi^2^	df	*P*		*p*_BA_
**All patients (*N*)**	374	476	–	–	–		–
Women	147 (39.3%)	228 (47.9%)	6.275	1	0.012		0.135
Men	227 (60.7%)	248 (52.1%)					
**Total number of admissions**	195 (52.1%)	293 (61.6%)	7.594	1	0.006		0.064^+^
**Age in years**	*M* = 43.4 SD = 17.9	*M* = 44.48 SD = 17.3	0.855^t^	848	0.393		1
**Time of presentation in the psychiatric emergency department**							
08:00–19:59	242 (64.7%)	349 (73.3%)	7.334	1	0.007		0.074^+^
20:00–07:59	132 (35.3%)	127 (26.7%)					
**Means of presentation**							
By their own means	186 (49.7%)	258 (54.2%)	1.802	1		0.179	1
Ambulance/police	188 (50.3%)	218 (45.8%)					
**Legal basis of psychiatric treatment**							
Voluntary	332 (88.8%)	427 (89.7%)	0.192	1	0.661		1
Involuntary	42 (11.2%)	49 (10.3%)					
**Re-presentation within 1 month**							
Yes	113 (30.2%)	97 (20.4%)	10.892	1	0.001		0.011^*^
No	261 (69.8%)	379 (79.6%)					
**Attempted suicide prior to presentation**							
Yes	12 (3.2%)	12 (2.5%)	0.361		0.548		1
No	362 (96.8%)	464 (97.5%)					
**Previous psychiatric treatment**							
No	72 (19.3%)	74 (15.5%)	2.265	2	0.132		1
Yes	283 (75.7%)	383 (80.5%)					
Unknown	21 (5.6%)	19 (4.0%)					

% of all patients presenting in the psychiatric emergency department in 2020 and 2019, respectively

*M* mean, *SD* standard deviation

^t^Value represents the *t* statistic

^+^Represents a trend < 0.1

*Represents a statistically significant finding < 0.05

Primary psychiatric diagnosis group of patients presenting in the PED differed significantly between 2019 and 2020 (*X*^2^ (5, *N* = 850) = 11.423, *p* = 0.044). Post hoc testing revealed that this is mostly due to a decrease in patients with affective disorders (ICD-10: F30–39). While in 2019 22.2% of patients presenting in the PED had a primary diagnosis of an affective disorder and therefore comprised the third largest diagnosis group, a significant decline to 15.2% was noted in 2020 (*X*^2^ (1, *N* = 163) = 6.675, *p* = 0.010). Further, more patients suffering from personality and behavioral disorders (ICD-10: F60–69) presented in the PED during 2020 (12.3 vs. 7.8%, *X*^2^ (1, *N* = 83) = 4.870, *p* = 0.027). Patients suffering from substance use disorders (ICD-10: F10–19) were the most common diagnosis group to utilize emergency psychiatric care (30.5% in 2020 and 29.0% in 2019) followed by patients suffering from schizophrenia, schizotypal, and delusional disorders (ICD: F20–29; 18.7% and 19.3%, respectively; Table 2), which will in short be referred to as “schizophrenia” in the following.

**Table 2:** Primary psychiatric diagnosis of patients presenting in the psychiatric emergency department in 2020 vs. 2019

	2020 (*N* = 374)	2019 (*N* = 476)	df	chi^2^	*p*	post hoc chi^2^	post hoc *p*
Substance use disorders (F10–19)	114 (30.5%)	138 (29.0%)	5	11.423	.044	0.223	0.637
Schizophrenia, schizotypal, and delusional disorders (F20–29)	70 (18.7%)	92 (19.3%)				0.051	0.822
Affective disorders (F30–39)	57 (15.2%)	106 (22.2%)				6.675	0.010^*^
Neurotic, stress-related, and somatoform disorders (F40–48)	70 (18.7%)	76 (16.0%)				1.271	0.291
Personality and behavioral disorders (F60–69)	46 (12.3%)	37 (7.8%)				4.870	0.027^*^
Others (F0, F50, F70–F90)	17 (4.5%)	27 (5.7%)				0.542	0.462

% of all patients presenting in the psychiatric emergency department in 2020 and 2019, respectively

*Represents a statistically significant finding < 0.05

Overall, PPA of patients presenting in the PED during 2020 showed significant differences when examining specific aspects. During the pandemic, patients were more likely to suffer from formal thought disorders (75.7 vs. 65.1%, *X*^2^ (1, *N* = 593) = 10.349, *p* = 0.001, *p*_BA_ = 0.030). While at both time points, most patients suffered from some type of affective disturbance (88.0 vs. 89.5%), significantly more patients stated a feeling of hopeless in 2020 (13.9 vs. 5.3%, *X*^2^ (1, *N* = 77) = 18.855, *p* < 0.000, *p*_BA_ < 0.000). Social withdrawal was significantly more prevalent in patients during the pandemic (14.4 vs. 8.0%, *X*^2^ (1, *N* = 92) = 11.334, *p* = 0.001, *p*_BA_ = 0.018). The rate of patients stating suicidal ideation (32.9 vs. 29.6%) and intent (12.3 vs. 9.9%) was stable (Table 3).

**Table 3:** Comparison of psychopathological assessment (PPA) categories of patients in 2020 vs.2019

	2020 (*N* = 374)	2019 (*N* = 476)	chi^2^	df	*p*	*p*_BA_
Disorientation	38 (10.2%)	50 (10.5%)	0.033	1	0.855	1
Cognitive disorder	195 (52.1%)	216 (45.4%)	3.860	1	0.049	1
Formal thought disorder	283 (75.7%)	310 (65.1%)	10.349	1	0.001	0.030^*^
Content thought disorder	91 (24.3%)	103 (21.6%)	0.698	1	0.403	1
Persecutory delusions	68 (18.2%)	60 (12.6%)	4.702	1	0.030	0.693
Fears and constraints	160 (42.8%)	158 (33.2%)	7.546	1	0.006	0.138
Anxiety	141 (37.7%)	134 (28.2%)	8.094	1	0.004	0.102
Compulsions	22 (5.9%)	14 (2.9%)	4.311	1	0.038	0.871
Delusions	58 (15.5%)	53 (11.1%)	3.284	1	0.070	1
Self-Disorder	43 (11.5%)	51 (10.7%)	0.082	1	0.775	1
Affective disturbance	329 (88.0%)	426 (89.5%)	1.055	1	0.304	1
Hopelessness	52 (13.9%)	25 (5.3%)	18.855	1	0.000	0.000 ^**^
Avolition	233 (62.3%)	326 (68.5%)	2.919	1	0.088	1
Social withdrawal	54 (14.4%)	38 (8.0%)	11.334	1	0.001	0.018^*^
Social impulsiveness	9 (2.4%)	13 (2.7%)	0.086	1	0.769	1
Aggressiveness	35 (9.4%)	34 (7.1%)	1.336	1	0.248	1
Self-harm	24 (6.4%)	37 (7.8%)	0.555	1	0.456	1
Lack of insight into illness	41 (11.0%)	53 (11.1%)	0.007	1	0.933	1
Suicidality	123 (32.9%)	142 (29.8%)	0.959	1	0.327	1
Suicidal ideation	123 (32.9%)	141 (29.6%)	1.095	1	0.295	1
Suicidal intent	46 (12.3%)	47 (9.9%)	0.025	1	0.876	1
Sleep disorders	93 (24.9%)	141 (29.6%)	0.208	1	0.648	1
Disruption of circadian rhythm	22 (5.9%)	21 (4.4%)	3.089	1	0.079	1

% of all patients presenting in the psychiatric emergency department in 2020 and 2019, respectively

*Represents a statistically significant finding < 0.05

**Represents a statistically significant finding < 0.01

### Mental and behavioral disorders due to psychoactive substance use (F10–19)

Both in 2020 and 2019, more male patients suffering from substance use disorders presented for emergency care (83.2% in 2020 vs. 64.5% in 2019; supplementary material Table 1). During the pandemic, patients with substance use disorders were more likely to be intoxicated with alcohol (76.3 vs. 64.5%, *X*^2^ (1, *N* = 252) = 7.646, *p* = 0.006, *p*_BA_ = 0.062). Blood/breath alcohol concentration (BAC) of these patients was higher in 2020 (*M* = 1.82, *SD* = 1.15‰) than in 2019 (*M* = 1.41, *SD* = 1.17‰, *t* (241) = − 2.727, *p*_BA_ = 0.076) indicating a trend of a more frequent consumption of higher amounts of alcohol. Suicidal ideation was stated significantly more often by patients with substance use disorders (47.4 vs. 26.8%, *X*^2^ (1, *N* = 252) = 12.650, *p* < 0.001, *p*_BA_ = 0.004) in 2020 than in 2019 (Table 4; supplementary material Table 1).

**Table 4:** Significant differences of characteristics within the different subgroups of primary psychiatric diagnosis in 2020 vs. 2019

	2020	2019	chi^2^	df	*p*	*p*_BA_
**Characteristics of patients with a primary diagnosis of ICD-10: F10–19;** **(*n***_**2020**_** = 114 vs. *n***_**2019**_** = 138)**						
Intoxication						
Patient is intoxicated with ≥ 1 substance	99 (86.8%)	103 (74.6%)	5.384	1	0.020	0.224
Intoxicated with alcohol	87 (76.3%)	89 (64.5%)	7.646	1	0.006	0.062^+^
BAC in ‰	*M* = 1.82 SD = 1.15	*M* = 1.41 SD = 1.17	− 2.727^t^	241	0.007	0.076^+^
Aspects of PPA						
Suicidal ideation	54 (47.4%)	37 (26.8%)	12.650	1	< 0.001	0.004^**^
**Characteristics of patients with a primary diagnosis of ICD-10: F20–29; (*n***_**2020**_ **= 70 vs.** ***n***_**2019**_** = 92)**						
Aspects of PPA						
Persecutory delusions	48 (68.7%)	40 (43.5%)	8.851	1	0.003	0.023^*^
Visual hallucinations	13 (18.6%)	3 (3.3%)	10.220	1	0.001	0.011^*^
**Characteristics of patients with a primary diagnosis of ICD-10: F30–39; (*n***_**2020**_** = 57 vs. *n***_**2019**_** = 106)**						
Re-presentation within 1 month	15 (26.3%)	11 (10.4%)	7.023	1	0.008	0.048^*^
**Characteristics of patients with a primary diagnosis of ICD-10: F40–48; (*n***_**2020**_** = 70 vs. *n***_**2019**_** = 76)**						
Men	34 (48.6%)	22 (28.9%)	5.935	1	0.015	0.060^+^
No prior psychiatric treatment	39 (55.7%)	28 (36.8%)	5.226	1	0.022	0.089^+^
**Characteristics of patients with a primary diagnosis of ICD-10: F60–69; (*n***_**2020**_** = 46 vs. *n***_**2019**_** = 37)**						
Men	18 (39.1%)	7 (18.9%)	3.980	1	0.046	0.322
Re-presentation within 1 month	24 (52.2%)	8 (21.6%)	8.080	1	0.004	0.031^*^
Resident of psychiatric residency	20 (43.5%)	4 (10.8%)	10.647	1	< 0.001	0.008^**^

% of all patients with primary diagnosis presenting in the psychiatric emergency department in 2020 and 2019, respectively

*BAC* blood/breath alcohol concentration, *M* mean, *SD* standard deviation, *PPA* psychopathological assessment

^t^Value represents the *t* statistic

^+^Represents a trend < 0.1

*Represents a statistically significant finding < 0.05

**Represents a statistically significant finding < 0.01

### Schizophrenia, schizotypal, and delusional disorders (F20–29)

Patients with schizophrenia presenting during the COVID-19 pandemic did not differ in most characteristics, such as gender, means of presentation, re-presentation, or suicidal ideation/intent (supplementary material Table 2). However, compared to the previous year, patients with schizophrenia were more likely to report persecutory delusions (68.7 vs. 43.5%, *X*^2^ (1, *N* = 162) = 8.851, *p* = 0.003, *p*_BA_ = 0.023) and visual hallucinations (18.6 vs. 3.3%, *X*^2^ (1, *N* = 162) = 10.220, *p* = 0.001, *p*_BA_ = 0.011) in 2020 (Table 4; supplementary material Table 2).

### Affective disorders (F30–39)

Patients suffering from affective disorders were significantly less likely to present in the PED in 2020 (15.2 vs. 22.2%, *X*^2^ (1, *N* = 163) = 6.675, *p* = 0.010; Table 2). At the same time, patients with affective disorders were significantly more likely to re-present within 1 month of previous psychiatric care than in 2019 (26.3 vs. 10.4%, *X*^2^ (1, *N* = 163) = 7.023, *p* = 0.008, *p*_BA_ = 0.048; Table 4; supplementary material Table 3).

### Neurotic, stress-related, and somatoform disorders (F40–48)

Patients suffering from neurotic, stress-related, and somatoform disorders presenting in the PED in 2020 were 1.7 times more likely to be male (48.6 vs. 28.9%, *X*^2^ (1, *N* = 146) = 5.935, *p* = 0.015, *p*_BA_ = 0.060). Also, proportionately more patients with neurotic, stress-related, and somatoform disorders, who had not received previous psychiatric treatment, presented in the PED during the pandemic (55.7 vs. 36.8%, *X*^2^ (1, *N* = 146) = 5.226, *p* = 0.022, *p*_BA_ = 0.089; Table 4; supplementary material Table 4). Both of these observations point towards a trend in changes among this patient group between 2019 and 2020.

### Personality and behavioral disorders (F60–69)

At both time points, patients within this diagnostic subgroup were most likely to suffer from emotionally unstable personality disorders (ICD-10: F60.30 and F60.31; 84.8% in 2020 vs. 91.9% in 2019; data not shown). Patients were significantly more likely to re-present in the PED within 1 month of previous treatment (52.5 vs. 21.6%, *X*^2^ (1, *N* = 83) = 8.080, *p* = 0.004, *p*_BA_ = 0.031). Furthermore, these patients were much more likely to live in a psychiatric residency in 2020 than 2019 (43.5 vs. 10.8%, *X*^2^ (1, *N* = 83) = 10.647, *p* < 0.001, *p*_BA_ = 0.008; Table 4; supplementary material Table 5).

### Association of mental health status with COVID-19

A total of *n* = 75 patients (20.1% of all patients presenting in the PED during the COVID-19 pandemic) reported a link between their mental health status and the current situation surrounding the COVID-19-pandemic. Patients suffering from neurotic, stress-related, and somatoform disorders most commonly stated an association to the COVID-19 pandemic (26.7% of *n* = 75 patients), followed by patients with substance use disorders (24.0%), affective disorders (20.0%), schizophrenia (18.7%), and personality and behavioral disorders (9.3%). Consequences of social measures such as “social distancing”, quarantine, and/or restrictions on leaving the house and visiting friends/family were named by 25 patients (33.3%) as a factor leading to a worsening of their mental health status, followed by changes in the structures of medical care (20 patients; 26.7%), and an increase or new onset of anxiety, fear, and/or compulsions (14 patients; 18.7%). Eight patients (10.7%) presented with delusional symptoms and/or hallucinations pertaining to COVID-19 and five patients (6.7%) stated that a loss of their job during the COVID-19 pandemic had led to a decline in mental health.

Suicidal ideation and intent were reported by 25 and 7 patients, respectively. Five patients (9.3% of patients stating an association with COVID-19) had attempted suicide prior to presentation in the PED. Thus, patients stating an association with COVID-19 were nearly three times more likely (OR = 2.9, *p* > 0.05) to have attempted suicide prior to presentation in the PED compared to the overall rate of suicide attempts leading to presentation in the PED of 3.2%.

## Discussion

Since the outbreak of severe acute respiratory syndrome coronavirus 2 (SARS-CoV-2), researchers have been examining the impact of the pandemic on mental health. Several authors have previously published results focusing on mental health of healthcare workers [9, 10] or the general population [11–14] derived from surveys and questionnaires. Main findings of these studies were that the COVID-19 crisis had great potential in destabilizing mental health, especially in regards to depressive and anxiety disorders [9, 11, 13]. However, questionnaires may not be a feasible tool to reach all psychiatric diagnosis groups such as patients with schizophrenia or substance use disorders.

The aim of this study was to detect the impact of the COVID-19-pandemic on patients within different psychiatric diagnostic subgroups presenting in the PED. The effect of the pandemic on psychiatric emergency presentations has been of interest to several authors who have conducted similar research in Portugal [15], Ireland [16], Western Australia [17], Norway [18], and Italy [19]. As in this study, these authors found a dramatic decrease in emergency visits ranging from 31 [16] to 52% [15] in temporal relation to a rising number of SARS-CoV-2 infections. Gonçalves-Pinho et al. found the greatest overall decline of emergency presentations and a decidedly greater relative decrease of 52.2% [15] in comparison to this study. A possible explanation for this may lie in the registered number of SARS-CoV-2 infections: By the endpoint of Gonçalves-Pinho et al.’s study on May 2nd, the region of Northern Portugal registered 4182 cases of SARS-CoV-2 per 10,000 residents [20, 21], which is 2.9 times higher than the number of cases registered in Lower Saxony at the respective endpoint of this study on May 24th (1450 cases of SARS-CoV-2 per 10,000 residents [21, 22]). The higher rate of infection may have resulted in a greater reluctance to seek medical care in Northern Portugal. Another expression of fear surrounding contracting an infection within the hospital setting may be that proportionately more patients came to the PED during the “off-hours” between 20:00 and 7:59 than in the previous year in hopes of reducing contact with others. Interestingly, McAndrew et al. made a similar observation in Ireland [16]. Furthermore, patients were twice as likely to leave the waiting area prior to contact with the psychiatrist on call. In Australia, the opposite observation was made with significantly less patients leaving prior to being attended [17].

A high frequency of repeat visits from psychiatric patients is a well-known phenomenon [23]. This study observed a significant increase of repeat visits within 1 month during the pandemic. This may be the result of tightened admission criteria as a response to the COVID-19 outbreak leading to an overall reduction of admissions to inpatient treatment in 2020, an observation also made by other psychiatric hospitals [15, 18]. A tightening of admission criteria and limiting inpatient treatment to “emergency only”, which was implemented by most hospitals [24, 25], may ultimately lead to an increase in unsatisfactory treatment outcomes due to premature discharge. Further, suggestions for ambulatory care are often made by the physician on call during emergency consultation, which, due to changed structures within the medical care system, may not have been feasible [24]. Consequently, the PED may have been the only option for timely psychiatric care [24].

Reviews on suicidal behavior during infectious disease-related public emergencies suggest that epidemics lead to an increased risk of death by suicides, though this evidence is currently supported by low-methodological quality studies [26, 27]. While one observational study detected an increase of suicidal ideation and behavior in emergency presentations [19], another found unaltered rates [16] as in this study, while other studies have even registered fewer suicide-related emergency presentations [17, 28] during the SARS-CoV-2 pandemic. This could suggest also that patients are less likely to seek care under these circumstances [16]. A limitation of this study is that it solely examined patients presenting for psychiatric care. Patients attempting suicide via intoxication or massive self-injury are more likely to present within other medical disciplines (i.e. internal medicine, trauma surgery), and therefore not included in this study due to the unavailability of this data. Patients presenting after attempted suicide were nearly three times more likely to associate their current mental health status with COVID-19. A recent study examining the link between COVID-19 and suicidal thoughts and behaviors suggested that nearly half of patients reporting suicidal ideation linked these thoughts to COVID-19 [29]. However, evidence supports that an increase of suicide rates is lagged by several months, as has been shown in the case of unemployment [30]. The extent of the pandemic’s true impact on suicide rates will become more apparent as time progresses.

While in Ireland substance use disorders, specifically of alcohol, were leading cause of emergency presentation in 2019, authors detected a significant decline in emergency presentations by these patients in 2020 [16], as did researchers in Australia [17]. In this study, first cause of emergency presentation in both 2019 and 2020 were substance use disorders. These patients presented with higher BAC which is in line with claims that the consumption of alcohol has increased during the SARS-CoV-2 pandemic [31]. Patients were also more likely to state suicidal ideation, which may be the result of destabilization of mental health due to social isolation [6], reduced outpatient support options [24] such as support groups, and a complete stop of elective alcohol detoxification. Interestingly, an increase in patients suffering from addiction/abuse of other substances, such as opioids and benzodiazepines, seeking emergency psychiatric care was not observed, even though a non-availability of these substances has been noted [29, 32]. This is in contrast to Dragovic et al. who noted an increased rate of drug-related presentations in Australia [17] and Capuzzi et al. who found an increase in PED visits by patients with cannabis use disorders in Italy [19].

Presentation rates of patients suffering from schizophrenia remained stable at slightly under 20% during both evaluated time periods. Merely small fluctuations of presentation rates within this diagnostic group have also been reported by others [15, 16, 19], while Dragovic et al. noted a decline [17]. In the present study, these patients were more likely to present with persecutory delusions and visual hallucinations during the pandemic. The plasticity of delusional content in relation to extrinsic factors is well-known [33], so it seems reasonable to assume that patients with schizophrenia may experience an exacerbation of psychotic symptoms, delusions, and/or fear reflecting the current situation [33, 34], as was the case in eight patients in this collective.

As previously observed by Gonçalves-Pinho et al., this study also found the most significant overall decrease in patients suffering from affective disorders seeking emergency psychiatric care during the pandemic. A decreased presentation rate of this patient group has been consistently reported by others [17, 19, 35]. At first glance this finding seems implausible considering a number of studies suggesting an increase in depressive disorders [13, 36, 37]. While this may have applied to the general population, those suffering from depressive disorders prior to the outbreak may have found a sense of stabilization brought on by certain measures of “social distancing” such as home office. Decreased emergency care utilization may point out that this diagnostic subgroup was well-served via telemedicine [38]. On the other hand, this study found that patients with affective disorders were more likely to re-present within 1 month of previous emergency psychiatric care, which may again point out insufficient outpatient treatment options.

During the pandemic, a surge in patients presenting with anxiety disorders was observed in Western Australia [17]. While it remained primary cause of presentation in Portugal, authors detected a slight decrease of patients with anxiety disorders in 2020 [15]. This study also noted an increase among this group of patients with neurotic, stress-related, and somatoform ranking as second most common cause of presentation in 2020 versus fourth in 2019. Interestingly, this study found a trend of more men within this diagnostic subgroup seeking emergency care during the COVID-19 pandemic compared to the previous year. This is contradictory to the assumption that women are more susceptible to experience a COVID-19-associated increase in anxiety [39]. In 2020, these patients were less likely to have received previous psychiatric treatment, which may point towards an increase of new onset of these disorders, which several authors have reported [13, 40, 41].

Patients suffering from personality and behavioral disorders showed significantly higher rates of re-presentation within 1 month during the 2020 pandemic relative to the previous year, especially among male patients. An increased utilization of emergency care by patients with personality and behavioral disorders was also registered in Portugal, however this applied predominantly to women [15]. In the present study, these patients were more likely to live in a psychiatric residency, which may indicate that this subgroup of patients is particularly susceptible to the impacts of a reduced availability of supportive measures such as group therapy, occupational therapy, etc. which were greatly reduced in order to adhere to social distancing policies. Likewise, patients living in psychiatric residential facilities showed increased emergency presentation rates in Italy [19].

This study detected an association between mental health and the pandemic in about one fifth of patients. COVID-19-related consultations were also noted in 22% of cases by Ness et al. in Norway [18]. It can be assumed that not all affected patients spontaneously commented on this aspect, therefore the number of patients negatively impacted by the pandemic is expected to be higher. In the event that an association between COVID-19 and a patient’s psychological well-being could be made, patients were most likely to state feeling particularly burdened by the consequences of social measures, which can take a severe toll on mental health both short- and long-term [6]. A limited availability of medical treatment, such as outpatient treatment, group therapy, day hospitals, or partial hospitalization after discharge, as well as restrictions within the inpatient as well as outpatient psychiatric care setting such as less face-to-face interaction and restrictions on communal dining, may lead to a decreased effectiveness of psychiatric treatment [24]. Psychiatric patients, especially chronically ill patients, dependent on these resources may be greatly de-stabilized by these shortcomings, leading them to utilize emergency care [24]. This raises the question to what extent the transmission risk of the activities limited by the implemented restrictions compares to the risk of transmission resulting from a visit in the emergency department as a consequence of these circumstances.

## Limitations

The results from this study should be interpreted in the context of its limitations. This study gathered data from a real-life emergency department setting. Apart from the physicians gathering data, the alternating psychiatrics on call were unaware of this study and therefore not instructed to explicitly ask and/or document how the COVID-19-pandemic and its implications were affecting a patient’s mental health. As a result, the actual rate of COVID-19 associated declines in psychological well-being may be much higher. On the other hand, it can be assumed that an association with the current situation surrounding the COVID-19 pandemic was only reported by the patient and documented by the physician in cases in which this was especially prevalent.

Information documented during PPA is both result of direct questioning by the treating physician, observation of the patient, as well as information spontaneously volunteered by the patient. The individual style of documentation of PPA varied in certain features between different psychiatrists (e.g. some did not routinely include sleeping disorders or circadian disturbances), however, most components considered relevant for this study were regularly assessed. Because of the emergency department setting and potential shortage of time, quality of PPA was occasionally lacking. This may have more often been the case in 2019 due to the higher number of patients frequenting the PED. Further, while the physicians on call continuously rotated both in 2019 and in 2020, the group of individual physicians differed between both time points. As a result, style of PPA may have varied further between 2019 and 2020. Moreover, while great efforts were taken to objectify data collection, confirmation bias cannot be fully ruled out, especially when the physicians performing data collection were on call.

PPA was assessed based solely on whether a certain characteristic applied to the patient or not. A quantification of these criteria was not performed due to insufficient information in regards to severity of symptoms in many PPAs. Therefore, this study only allows for a comparison of patients presenting with or without a certain characteristic of PPA but does not allow an examination of how pronounced that characteristic was. This may have limited the significance of certain findings that are hallmarks of specific psychiatric diagnosis such as anxiety among patients with anxiety disorders or chronic suicidal ideation and self-harm in patients with emotionally unstable personality disorder.

Perhaps the greatest limitation of this study is that only two time points were compared (i.e. 2019 and 2020). It therefore cannot be excluded that patient data is highly variable as a rule and shows disparate trends between timeframes in general. This consequently limits statistical contextualization of the results presented here. Further, this study had a monocentric design—other psychiatric emergency departments may observe a different constellation of patients presenting for emergency care.

## Conclusion and clinical implications

This study shows the immediate effects of the COVID-19 pandemic on mental health in patients seeking emergency psychiatric care in Germany. By understanding how different patient groups are impacted by the pandemic and its implications on everyday life, we can begin to comprehend which deficits our health system is faced with. This is the first step to improving structures within all settings of psychiatric care including inpatient and outpatient treatment and psychiatric residencies, to be able to provide  optimized health care services in the future.

## Supplementary Information

Below is the link to the electronic supplementary material.

Supplementary file1 (DOCX 37 KB)

## Data Availability

Upon reasonable request.

## References

[1] Cucinotta D, Vanelli M (2020). WHO declares COVID-19 a pandemic. Acta Biomed.

[2] Shah K, Kamrai D, Mekala H, Mann B, Desai K, Patel RS (2020). Focus on mental health during the coronavirus (COVID-19) pandemic: applying learnings from the past outbreaks. Cureus.

[3] Carvalho LF, Pianowski G, Gonçalves AP (2020). Personality differences and COVID-19: are extroversion and conscientiousness personality traits associated with engagement with containment measures?. Trends Psychiatry Psychother.

[4] Shigemura J, Ursano RJ, Morganstein JC, Kurosawa M, Benedek DM (2020). Public responses to the novel 2019 coronavirus (2019-nCoV) in Japan: mental health consequences and target populations. Psychiatry Clin Neurosci.

[5] Menninger KA (1919). Psychoses associated with influenza, I: general data: statistical analysis. JAMA.

[6] Brooks SK, Webster RK, Smith LE, Woodland L, Wessely S, Greenberg N, Rubin GJ (2020). The psychological impact of quarantine and how to reduce it: rapid review of the evidence. Lancet.

[7] World Health Organization (1992) The ICD-10 classification of mental and behavioural disorders

[8] AMDP (2018). Das AMDP-System: Manual zur Dokumentation psychiatrischer Befunde.

[9] Li Z, Ge J, Yang M, Feng J, Qiao M, Jiang R, Bi J, Zhan G, Xu X, Wang L, Zhou Q, Zhou C, Pan Y, Liu S, Zhang H, Yang J, Zhu B, Hu Y, Hashimoto K, Jia Y, Wang H, Wang R, Liu C, Yang C (2020). Vicarious traumatization in the general public, members, and non-members of medical teams aiding in COVID-19 control. Brain Behav Immun.

[10] Kang L, Ma S, Chen M, Yang J, Wang Y, Li R, Yao L, Bai H, Cai Z, Xiang Yang B, Hu S, Zhang K, Wang G, Ma C, Liu Z (2020). Impact on mental health and perceptions of psychological care among medical and nursing staff in Wuhan during the 2019 novel coronavirus disease outbreak: a cross-sectional study. Brain Behav Immun.

[11] Wang C, Pan R, Wan X, Tan Y, Xu L, Ho CS, Ho RC (2020). Immediate psychological responses and associated factors during the initial stage of the 2019 coronavirus disease (COVID-19) epidemic among the general population in China. Int J Environ Res Public Health.

[12] Moccia L, Janiri D, Pepe M, Dattoli L, Molinaro M, De Martin V, Chieffo D, Janiri L, Fiorillo A, Sani G, Di Nicola M (2020). Affective temperament, attachment style, and the psychological impact of the COVID-19 outbreak: an early report on the Italian general population. Brain Behav Immun.

[13] Huang Y, Zhao N (2020). Generalized anxiety disorder, depressive symptoms and sleep quality during COVID-19 outbreak in China: a web-based cross-sectional survey. Psychiatry Res.

[14] Qiu J, Shen B, Zhao M, Wang Z, Xie B, Xu Y (2020). A nationwide survey of psychological distress among Chinese people in the COVID-19 epidemic: implications and policy recommendations. Gen Psychiatr.

[15] Gonçalves-Pinho M, Mota P, Ribeiro J, Macedo S, Freitas A (2020). The impact of COVID-19 pandemic on psychiatric emergency department visits—a descriptive study. Psychiatr Q.

[16] McAndrew J, O’Leary J, Cotter D, Cannon M, MacHale S, Murphy K, Barry H (2020). Impact of initial COVID-19 restrictions on psychiatry presentations to the emergency department of a large academic teaching hospital. Ir J Psychlog Med.

[17] Dragovic M, Pascu V, Hall T, Ingram J, Waters F (2020). Emergency department mental health presentations before and during the COVID-19 outbreak in Western Australia. Australas Psychiatry.

[18] Ness E, Salvador EM, Gardsjord ES (2020). Patient visits to a psychiatric casualty clinic during the initial phase of the COVID-19 pandemic. Tidsskr Nor Laegeforen.

[19] Capuzzi E, Di Brita C, Caldiroli A, Colmegna F, Nava R, Buoli M, Clerici M (2020). Psychiatric emergency care during Coronavirus 2019 (COVID 19) pandemic lockdown: results from a Department of Mental Health and Addiction of northern Italy. Psychiatry Res.

[20] Instituto Nacional de Estatistica (2020) https://www.ine.pt/xportal/xmain?xpid=INE&xpgid=ine_indicadores&contecto=pi&indOcorrCod=0008273&selTab=tab0. Accessed 28 Oct 2020

[21] Direção-Geral da Saúde (2020) https://covid19.min-saude.pt/relatorio-de-situacao/. Accessed 28 Oct 2020

[22] Robert Koch Institut (2020) https://www.rki.de/DE/Content/InfAZ/N/Neuartiges_Coronavirus/Fallzahlen.html. Accessed 28 Oct 2020

[23] Juhás M, Agyapong VI (2016). Patients assessed by the liaison psychiatric team in the emergency department of a regional hospital in Canada—general characteristics and gender differences. Int J Psychiatry Clin Pract.

[24] Bojdani E, Rajagopalan A, Chen A, Gearin P, Olcott W, Shankar V, Cloutier A, Solomon H, Naqvi NZ, Batty N, Festin FED, Tahera D, Chang G, DeLisi LE (2020). COVID-19 pandemic: impact on psychiatric care in the United States. Psychiatry Res.

[25] Starace F, Ferrara M (2020). COVID-19 disease emergency operational instructions for Mental Health Departments issued by the Italian Society of Epidemiological Psychiatry. Epidemiol Psychiatr Sci.

[26] Leaune E, Samuel M, Oh H, Poulet E, Brunelin J (2020). Suicidal behaviors and ideation during emerging viral disease outbreaks before the COVID-19 pandemic: a systematic rapid review. Prev Med.

[27] Zortea TC, Brenna CTA, Joyce M, McClelland H, Tippett M, Tran MM, Arensman E, Corcoran P, Hatcher S, Heise MJ, Links P, O'Connor RC, Edgar NE, Cha Y, Guaiana G, Williamson E, Sinyor M, Platt S (2020). The impact of infectious disease-related public health emergencies on suicide, suicidal behavior, and suicidal thoughts. Crisis.

[28] Hernández-Calle D, Martínez-Alés G, Mediavilla R, Aguirre P, Rodríguez-Vega B, Bravo-Ortiz MF (2020). Trends in psychiatric emergency department visits due to suicidal ideation and suicide attempts during the COVID-19 pandemic in Madrid, Spain. J Clin Psychiatry.

[29] Chatterjee SS, Barikar CM, Mukherjee A (2020). Impact of COVID-19 pandemic on pre-existing mental health problems. Asian J Psychiatr.

[30] Nordt C, Warnke I, Seifritz E, Kawohl W (2015). Modelling suicide and unemployment: a longitudinal analysis covering 63 countries, 2000–11. Lancet Psychiatry.

[31] Da BL, Im GY, Schiano TD (2020). Coronavirus disease 2019 hangover: a rising tide of alcohol use disorder and alcohol-associated liver disease. Hepatology.

[32] United Nations (2020) COVID-19 is changing the route of illict drug flows, says UNODC report. https://www.unodc.org/unodc/en/press/releases/2020/May/covid-19-is-changing-the-route-of-illicit-drug-flows--says-unodc-report.html. Accessed 1 Jun 2020

[33] Campbell MM, Sibeko G, Mall S, Baldinger A, Nagdee M, Susser E, Stein DJ (2017). The content of delusions in a sample of South African Xhosa people with schizophrenia. BMC Psychiatry.

[34] Maguire PA, Reay RE, Looi JC (2019). A sense of dread: affect and risk perception in people with schizophrenia during an influenza pandemic. Australas Psychiatry.

[35] Hoyer C, Ebert A, Szabo K, Platten M, Meyer-Lindenberg A, Kranaster L (2020). Decreased utilization of mental health emergency service during the COVID-19 pandemic. Eur Arch Psychiatry Clin Neurosci.

[36] Zhang J, Lu H, Zeng H, Zhang S, Du Q, Jiang T, Du B (2020). The differential psychological distress of populations affected by the COVID-19 pandemic. Brain Behav Immun.

[37] Zhang SX, Wang Y, Rauch A, Wei F (2020). Unprecedented disruption of lives and work: health, distress and life satisfaction of working adults in China one month into the COVID-19 outbreak. Psychiatry Res.

[38] Kolar D (2020). Psychiatric emergency services and non-acute psychiatric services utilization during COVID-19 pandemic. Eur Arch Psychiatry Clin Neurosci.

[39] Özdin S, Bayrak Özdin Ş (2020). Levels and predictors of anxiety, depression and health anxiety during COVID-19 pandemic in Turkish society: the importance of gender. Int J Soc Psychiatry.

[40] Banerjee DD (2020). The other side of COVID-19: Impact on obsessive compulsive disorder (OCD) and hoarding. Psychiatry Res.

[41] Bhatia MS, Goyal S, Singh A, Daral A (2020). COVID-19 pandemic-induced panic disorder. Prim Care Companion CNS Disord.

