# Supplementary material for: Peripandemic psychiatric emergencies: impact of the COVID-19 pandemic on patients according to diagnostic subgroup
Source: Eur Arch Psychiatry Clin Neurosci. 2021 Feb 5;271(2):259–70. doi: 10.1007/s00406-020-01228-6 (PMC7862867; doi:10.1007/s00406-020-01228-6)
Supplement: Supplementary file 1 — Supplementary file1 (DOCX 37 KB) [file 406_2020_1228_MOESM1_ESM.docx]

**Supplementary Material**

**Peripandemic psychiatric emergencies:** impact of the COVID-19 pandemic on patients according to diagnostic subgroup

Johanna Seifert^1^, Catharina Meissner^1^, Anna Birkenstock^1^, Stefan Bleich^1^, Sermin Toto^1^, Christian Ihlefeld^1^, Tristan Zindler^1^

^1^ Department of Psychiatry, Social Psychiatry and Psychotherapy, Hannover Medical School, Germany

**Corresponding author** Dr. med. Johanna Seifert

E-Mail: seifert.johanna@mh-hannover.de

**Supplementary Material Table 1:** Characteristics of patients with a primary diagnosis of substance use disorders (ICD-10: F10–19)

|  | **2020 (N=114)** | **2019 (N=138)** | **chi²** | **df** | ***p*** | ***p*_BA_** |
| --- | --- | --- | --- | --- | --- | --- |
| **Men** | 95 (83.2%) | 104 (75.4%) | 2.388 | 1 | .122 | 1 |
| **Intoxication** | | | | | | |
| No | 15 (13.2%) | 34 (24.6%) | 5.384 | 1 | .020 | .224 |
| Yes | 99 (86.8%) | 103 (74.6%) |  |  |  |  |
| 1 substance | 91 (79.8%) | 91 (65.9%) | 1.192 | 1 | .2749 | 1 |
| ≥ 2 substances | 7 (6.1%) | 12 (8.7%) |  |  |  |  |
| Intoxicated with alcohol | 87 (76.3%) | 89 (64.5%) | 7.646 | 1 | .006 | **.062^+^** |
| BAC in **‰** | *M* = 1.82  *SD* = 1.15 | *M* =1.41  *SD* = 1.17 | -2.727^t^ | 241 | .007 | **.076^+^** |
| **Specific diagnosis** | | | | | | |
| Use of alcohol (F10) | 94 (82.5%) | 103 (74.6%) | 2.237 | 1 | .1348 | .120 |
| Use of other substances  (F11–19) | 20 (17.5%) | 35 (25.4%) |  |  |  | 1 |
| **Means of presentation** | | | | | | |
| By their own means | 27 (23.7%) | 51 (37.0%) | 4.949 | 1 | .026 | .287 |
| Ambulance/police | 86 (75.4%) | 87 (63.0%) |  |  |  |  |
| **Re-presentation** | | | | | | |
| Within 1 month | 42 (36.8%) | 41 (29.7%) | 1.438 | 1 | .231 | 1 |
| **Aspects of PPA** | | | | | | |
| Suicidal ideation | 54 (47.4%) | 37 (26.8%) | 12.650 | 1 | <.001 | **.004^**^** |
| Suicidal intent | 21 (21.1%) | 24 (17.3%) | 1.704 | 1 | .192 | 1 |
| Aggressiveness | 20 (17.5%) | 10 (7.2%) | 6.312 | 1 | .012 | .132 |

% of all patients with a primary diagnosis of ICD-10: F10-19 presenting in the psychiatric emergency department in 2020 and 2019, respectively

BAC: blood/breath alcohol concentration; M: mean; SD: standard deviation; PPA: psychopathological assessment

^t^ value represent the t-statistic
^+^ represents a trend <.1, * represents a statistically significant finding <.05, ** represents a statistically significant finding <.01

**Supplementary Material Table 2:** Characteristics of patients with a primary diagnosis of schizophrenia, schizotypal, and delusional disorders (ICD-10: F20–29)

|  | **2020 (N=70)** | **2019 (N=92)** | **chi²** | **df** | ***p*** | ***p*_BA_** |
| --- | --- | --- | --- | --- | --- | --- |
| **Men** | 45 (64.3%) | 55 (59.8%) | 0.341 | 1 | .559 | 1 |
| **Means of presentation** | | | | | | |
| By their own means | 38 (54.3%) | 52 (56.6%) | 0.080 | 1 | .777 | 1 |
| Ambulance/police | 32 (45.7%) | 40 (43.5%) |  |  |  | 1 |
| **Re-presentation** | | | | | | |
| Within 1 month | 23 (32.9%) | 21 (22.8%) | 2.022 | 1 | .155 | 1 |
| **Aspects of PPA** | | | | | | |
| Suicidal ideation | 12 (17.1%) | 13 (14.1%) | 0.226 | 1 | .634 | 1 |
| Suicidal intent | 6 (8.6%) | 3 (3.3%) | 2.052 | 1 | .152 | 1 |
| Persecutory delusions | 48 (68.7%) | 40 (43.5%) | 8.851 | 1 | .003 | **.023^*^** |
| Auditory hallucinations | 28 (40.0%) | 23 (25.0%) | 3.862 | 1 | .049 | .395 |
| Visual hallucinations | 13 (18.6%) | 3 (3.3%) | 10.220 | 1 | .001 | **.011^*^** |

% of all patients with a primary diagnosis of ICD-10: F20-29 presenting in the psychiatric emergency department in 2020 and 2019, respectively

PPA: psychopathological assessment

^+^ represents a trend <.1, * represents a statistically significant finding <.05, ** represents a statistically significant finding <.01

**Supplementary Material Table 3:** Characteristics of patients with a primary diagnosis of affective disoders (ICD-10: F30–39)

|  | **2020 (N=57)** | **2019 (N=106)** | **chi²** | **df** | ***p*** | ***p*_BA_** |
| --- | --- | --- | --- | --- | --- | --- |
| **Men** | 25 (39.5%) | 53 (50.0%) | 0.560 | 1 | .454 | 1 |
| **Specific diagnosis** | | | | | | |
| Manic and bipolar disorders (F30–31) | 19 (33.3%) | 20 (18.9%) | 4.261 | 1 | .039 | .234 |
| MDD (F32–33) | 38 (66.7%) | 86 (81.1%) |  |  |  |  |
| **Means of presentation** | | | | | | |
| By their own means | 38 (66.7%) | 73 (68.9%) | 0.140 | 1 | .708 | 1 |
| Ambulance/police | 19 (33.3%) | 32 (30.2%) |  |  |  |  |
| **Re-presentation** | | | | | | |
| Within 1 month | 15 (26.3%) | 11 (10.4%) | 7.023 | 1 | .008 | **.048^*^** |
| **Aspects of PPA** | | | | | | |
| Suicidal ideation | 21 (36.8%) | 48 (45.3%) | 1.082 | 1 | .298 | 1 |
| Suicidal intent | 8 (14.0%) | 21 (19.8%) | 0.846 | 1 | .358 | 1 |

% of all patients with a primary diagnosis of ICD-10: F30-39 presenting in the psychiatric emergency department in 2020 and 2019, respectively

MDD: major depressive disorder; PPA: psychopathological assessment

^+^ represents a trend <.1, * represents a statistically significant finding <.05, ** represents a statistically significant finding <.01

**Supplementary Material Table 4:** Characteristics of patients with a primary diagnosis of neurotic, stress-related, and somatoform disorders (ICD-10: F40–48)

|  | **2020 (N=70)** | **2019 (N=76)** | **chi²** | **df** | ***p*** | ***p*_BA_** |
| --- | --- | --- | --- | --- | --- | --- |
| **Men** | 34 (48.6%) | 22 (28.9%) | 5.935 | 1 | .015 | **.060^+^** |
| **Prior psychiatric treatment** | | | | | | |
| Yes | 31 (44.3%) | 48 (63.2%) | 5.226 | 1 | .022 | **.089^+^** |
| No | 39 (55.7%) | 28 (36.8%) |  |  |  |  |
| **Specific diagnosis** | | | | | | |
| Phobic, anxiety disorders, OCD (F40-42) | 31 (44.3%) | 26 (34.2%) | 9.454 | 4 | .051 | .203 |
| Acute stress reaction/adjustment disorder (F43.0, F43.2) | 17 (24.3%) | 25 (32.9%) |  |  |  |  |
| Posttraumatic stress disorder (PTSD; F43.1) | 14 (20.0%) | 7 (9.2%) |  |  |  |  |
| Somatoform disorders (F45) | 7 (10.0%) | 11 (14.5%) |  |  |  |  |
| Others (F44, F48) | 1 (1.4%) | 7 (9.2%) |  |  |  |  |
| **Re-presentation** | | | | | | |
| Within 1 month | 7 (10.0%) | 13 (17.1%) | 1.556 | 1 | .212 | .849 |

% of all patients with a primary diagnosis of ICD-10: F40-48 presenting in the psychiatric emergency department in 2020 and 2019, respectively

OCD: obsessive compulsive disorder; PTSD: posttraumatic stress disorder

^+^ represents a trend <.1, * represents a statistically significant finding <.05, ** represents a statistically significant finding <.01

**Supplementary Material Table 5:** Characteristics of patients with a primary diagnosis of personality and behavioral disorders (ICD-10: F60–69)

|  | **2020 (N=46)** | **2019 (N=37)** | **chi²** | **df** | ***p*** | ***p*_BA_** |
| --- | --- | --- | --- | --- | --- | --- |
| **Men** | 18 (39.1%) | 7 (18.9%) | 3.980 | 1 | .046 | **.322** |
| **Re-presentation** | | | | | | |
| Within 1 month | 24 (52.2%) | 8 (21.6%) | 8.080 | 1 | .004 | **.031^*^** |
| **Living situation** | | | | | | |
| Psychiatric residency | 20 (43.5%) | 4 (10.8%) | 10.647 | 1 | <.001 | **.008^**^** |
| Others | 26 (56.5%) | 33 (89.2%) |  |  |  |  |
| **Aspects of PPA** | | | | | | |
| Suicidal ideation | 22 (47.8%) | 25 (67.6%) | 2.127 | 1 | .049 | .347 |
| Suicidal intent | 3 (6.5%) | 6 (16.2%) | 3.248 | 1 | .145 | 1 |
| Anxiety | 11 (23.9%) | 2 (5.4%) | 4.889 | 1 | .027 | .190 |
| Self-harm | 14 (30.4%) | 18 (48.6%) | 3.249 | 1 | .071 | .500 |

% of all patients with a primary diagnosis of ICD-10: F60–69 presenting in the psychiatric emergency department in 2020 and 2019, respectively

PPA: psychopathological assessment

^+^ represents a trend <.1, * represents a statistically significant finding <.05, ** represents a statistically significant finding <.01
